# The mitochondrial brain: From mitochondrial genome to neurodegeneration

**DOI:** 10.1016/j.bbadis.2009.07.010

**Published:** 2010-01

**Authors:** Helen E. Turnbull, Nichola Z. Lax, Daria Diodato, Olaf Ansorge, Doug M. Turnbull

**Affiliations:** a Mitochondrial Research Group, Institute for Ageing and Health, Newcastle University, NE2 4HH, UK; b Newcastle University Centre for Brain Ageing and Vitality, Institute for Ageing and Health, Newcastle University, NE2 4HH, UK; c Department of Neuropathology, John Radcliffe Hospital, Oxford, OX3 9DU, UK

**Keywords:** Mitochondrial DNA

## Abstract

Mitochondrial DNA mutations are an important cause of neurological disease. The clinical presentation is very varied in terms of age of onset and different neurological signs and symptoms. The clinical course varies considerably but in many patients there is a progressive decline, and in some evidence of marked neurodegeneration. Our understanding of the mechanisms involved is limited due in part to limited availability of animal models of disease. However, studies on human post-mortem brains, combined with clinical and radiological studies, are giving important insights into specific neuronal involvement.

## 1 Introduction

Mitochondrial DNA (mtDNA) mutations are increasingly becoming recognised as important causes of disease. They were first identified in 1988 [1,2] and were initially thought to be relatively rare, however, the incidence of known pathogenic mutations in the general population has recently been investigated. These studies have shown that approximately 1 in 500 individuals carry the m.3243A>G mutation [3], which can cause severe neurological disease, with a similar figure observed for the m.1555A>G mutation, which causes aminoglycoside-induced deafness [4,5]. Another approach to study the incidence of mtDNA disease is to document the number of clinically affected cases within a specific geographic region. This approach is limited because there is marked clinical variability which means many patients go unrecognised and because patients may not be referred to specialist centres. However, even these studies show there is a high disease burden with at least 1 in 10,000 of the adult population suffering from mtDNA disease [6].

In the twenty years since mtDNA mutations were first associated with human disease it has become apparent that the central nervous system is one of the predominant systems affected, especially in those patients with a severe phenotype. Despite this we only have limited understanding of the mechanisms of neurodegeneration. One thing that has become clear, however, is that certain mtDNA mutations have predilections for causing neurodegeneration in certain areas of the brain.

Patients with mtDNA disease present with a plethora of neurological manifestations, in particular, optic atrophy, ataxia, seizures, progressive weakness, dementia, stroke-like episodes and extrapyramidal features. The unique nature of mitochondrial genetics means that patients with the same mutation may have very different clinical presentations. This highlights the complexity of clinical phenotyping, and the importance of understanding the underlying pathogenesis and developing treatments for patients with mtDNA disease.

In addition to the increasing number of patients being recognised with mtDNA disease, mtDNA mutations have also been found to be present in high levels in several ageing tissues. For example, mtDNA mutations have been identified in the neurons of the substantia nigra in both elderly control subjects and in patients with Parkinson's disease [7,8]. There is a direct link between the presence of these mutations and a mitochondrial defect in these cells. Thus a greater understanding of disease mechanisms in patients with mtDNA disease may also give important insights into neuronal loss or dysfunction in ageing and age-related neurodegenerative diseases [9–11].

This review focuses entirely on the neurological and neuropathological features of patients with mtDNA disease. Mitochondrial disease can also be caused by nuclear gene disorders [12], many of which mimic the features seen in mtDNA disease, but these are not discussed here. We first review important aspects of mitochondrial genetics, crucial if we are to understand the pathogenesis of the changes seen. We then describe the clinical features seen in patients and the associated neuropathology. Unfortunately at present there is limited treatment available for patients with mtDNA mutations and we discuss various potential therapies.

## 2 Mitochondria — structure and function

Mitochondria are double-membrane subcellular organelles which were originally primitive, autonomous, bacteria-like organisms. During evolution these organisms were engulfed by larger eukaryotic cells; enjoying protection in return for providing energy. The relationship was so successful that we are now dependent on mitochondria for over 90% of our cellular energy in the form of ATP generated by the process of oxidative phosphorylation. This endosymbiotic relationship is almost certainly the origin of the mitochondrial genome which rather surprisingly has been maintained throughout evolution [13].

Mitochondrial function is closely linked to its unique double-membrane structure. The outer mitochondrial membrane is porous to small molecular weight substances and encapsulates the mitochondria. The enzymes of the mitochondrial respiratory chain are found within the highly folded inner membrane which separates the matrix from the intermembrane space. The matrix contains the mitochondrial genome, as well as the enzymes involved in the tricarboxylic acid cycle and mitochondrial fatty acid oxidation. The cristae, or finger-like projections, of the inner mitochondrial membrane ensure the maximum surface area between the biochemical substrates in the matrix and the respiratory chain enzymes. The inner membrane is also highly impermeable allowing the free passage of only water, oxygen and carbon dioxide.

The primary function of mitochondria is the generation of ATP from ADP by the process of oxidative phosphorylation. This process is dependent upon five multisubunit complexes (I–V), only one of which (complex II) is totally encoded by the nuclear genome. All the other complexes have subunits encoded by both mitochondrial and nuclear genomes, making them vulnerable to mutations of either genome. Through a succession of oxidoreduction reactions, electrons are transported along the respiratory chain to molecular oxygen. This generates an electrochemical gradient by the extrusion of protons. This gradient then drives ATP synthesis by complex V (ATP synthase).

Mitochondria are often considered as discrete organelles however this is not the case with mitochondria often forming networks within cells. There is constant fission and fusion of mitochondria and if this process is disrupted then mitochondrial disease develops and interestingly accumulation of mutations [14]. The fission and fusion process is also thought to be important in targeting mitochondria for turnover by the process of autophagy [15].

## 3 Mitochondrial genetics and biology

### 3.1 The mitochondrial genome

Mitochondrial DNA was identified in 1963. It has subsequently been shown that multiple copies of this double-stranded, super-coiled, circular molecule are found within the matrix of mitochondria. There are between 2 and 10 mtDNA copies per mitochondrion, resulting in 1000–100,000 copies per cell. This is the only extra chromosomal DNA in human cells. The mitochondrial genome consists of 16,569 base pairs and the entire mtDNA sequence was first reported in 1981 [16] and is referred to as the Cambridge reference sequence. This has since been revised to eliminate errors caused by the use of bovine fragments in the original sequencing project [17].

The mitochondrial genome encodes 13 essential respiratory chain subunits as well as the genetic information for the coding of 2 ribosomal RNAs (rRNA) and 22 transfer RNAs (tRNA) required for the intramitochondrial sequence of these proteins. The majority of mitochondrial proteins are nuclear encoded and these proteins form the other subunits of the respiratory chain as well those required for mtDNA maintenance. Thus nuclear gene mutations of the proteins involved in mtDNA replication and/or repair will have profound effects on the mitochondrial genome.

The mitochondrial genome seems to be particularly vulnerable to damage. It has been proposed that the mutation rate is at least 10 times greater than that of the nuclear genome and there are a number of possible reasons for this. The mtDNA lacks protective histones and has limited repair mechanisms. It is also susceptible to nucleolytic attack from the free radicals produced by oxidative phosphorylation. Combined with this the mtDNA consists entirely of exons and consequently there is very little redundancy. As a result a point mutation or deletion can very quickly lead to a biochemical defect.

### 3.2 MtDNA replication, transcription and translation

The two strands of the mitochondrial genome differ in their distribution of bases C and G making one strand denser (called the heavy or H strand) than the other (light or L strand). There is a non-coding control region of about 1.1 kb which is often referred to as the D-loop. This region contains essential sequences for initiation of replication and transcription.

#### 3.2.1 MtDNA replication

Individual mtDNA can replicate independently of the cell cycle and is therefore described as relaxed. Clayton originally described mtDNA replication as an asynchronous mechanism originating from two points O_H_ and O_L_ [18]. An RNA primer generated from the light chain initiates replication of an mtDNA molecule at the origin of the heavy chain, O_H_, found in the D-loop. DNA polymerase gamma, encoded by a nuclear gene, then synthesises the DNA strand. The origin for the light chain, O_L_, is a small non-coding region surrounded by tRNA genes. It is exposed when the heavy strand passes this region, about 2/3 of its way around the genome and L-strand replication is initiated in the opposite direction.

Recently a strand-coupled method has been proposed in which the two strands are synthesised simultaneously [19]. In this model of mtDNA replication, lagging L-strand synthesis starts shortly after the initiation of replication at O_H_ and may involve extensive RNA synthesis prior to DNA synthesis. However, there remains debate as to which form of mtDNA replication predominates and even whether different forms of replication occur in different tissues.

#### 3.2.2 MtDNA transcription

There is less contention as regards mtDNA transcription and many of the proteins involved have been identified and an *in vitro* system established. Mitochondrial transcription is initiated from promoters on both H and L-strand generating polycistronic transcripts, which are then processed to produce the individual mRNA, rRNA and tRNA molecules. This process requires a mitochondrial RNA polymerase, a transcription activator called TFAM, and either mitochondrial transcription factor B1 or B2 [20].

#### 3.2.3 MtDNA translation

Mitochondrial translation is still an area in which there are many uncertainties. It is a process controlled by nuclear encoded proteins [21] which include two specific mitochondrial initiation factors [22], three mitochondrial elongation factors [23] and at least one termination release factor [24].

### 3.3 Mitochondrial DNA mutations

The first pathogenic mtDNA mutations were described in 1988 and since then over 300 mtDNA mutations have been reported (Mitomap) [25]. These mutations take the form of either point mutations or rearrangements (deletions or duplications). They result in a biochemical defect either by affecting protein synthesis if the sequence of a tRNA or rRNA is disrupted; or, the normal functioning of the respiratory chain if one of the genes encoding the subunit is affected. There are several important features unique to the mitochondrial genome when we consider its role in human disease.

#### 3.3.1 Homoplasmy and heteroplasmy

There are multiple copies of the mitochondrial genome in all cells. The normal situation is that all the copies of mtDNA within the cell are identical and have wild-type mtDNA sequence. In the presence of an mtDNA mutation all copies of the mitochondrial genome may be affected which is known as a homoplasmic mutation. Most pathogenic mutations only affect a proportion of the mitochondrial genomes and this is called heteroplasmy.

#### 3.3.2 Threshold effect

In the presence of a heteroplasmic mtDNA defect not all cells show evidence of a biochemical defect, observed as a mosaic of cells lacking mitochondrial enzyme activity, often as cytochrome *c* oxidase deficiency [26]. This is due to a threshold effect with mitochondrial function only being affected if there are high levels of mutated mtDNA within individual cells [27]. It is thought that the critical factor is the amount of wild-type mtDNA present to complement the defective mtDNA. Typically between 70 and 90% mutated mtDNA is required for the clinical phenotype to develop [28], but this varies markedly for different mtDNA mutations. The threshold level also appears to vary for different cell types and this may explain why some tissues are more affected in the presence of some mtDNA mutations than others.

#### 3.3.3 Mitotic segregation

In dividing cells not all daughter cells will contain the same level of mutated mtDNA. In cells undergoing mitotic division the cells with high levels of mtDNA mutation appear to be at a disadvantage and the mtDNA mutation is lost during life [29]. In post-mitotic cells such as muscle and neurons there cannot be loss of the mutated mtDNA by mitotic segregation and this may be a factor in their frequent involvement in mitochondrial disease.

#### 3.3.4 Maternal transmission and bottleneck

Numerous population based genetic studies have shown that the transmission of mtDNA is purely maternal. Indeed the uniparental inheritance of mtDNA, as well as the high rate of mutation, has made mtDNA invaluable in charting the evolution of man [30]. Maternal inheritance is also very important in the context of mitochondrial diseases. Large maternal pedigrees have been identified, although sometimes there are apparently unaffected family members. This may be due to variation in the expression of the disease or in the presence of low levels of heteroplasmic mutations. Heteroplasmic mutations can vary widely between offspring due to the presence of a mitochondrial bottleneck during development. The copy number of mtDNA molecules in primordial germ cells falls to very low levels [31–33]. This bottleneck has a crucial role in preventing the transmission of very deleterious mutations. This purifying selection seems to occur during the cell replication phase in primordial germ cells. The stage in the process which differences in heteroplasmy between offspring occur is less certain, but it is a major feature in many families with heteroplasmic mtDNA disease.

## 4 Possible mechanisms of neurodegeneration in mtDNA disease

Despite research to elucidate the specific pathological mechanisms leading to cell loss in mtDNA disease our understanding remains limited. Compromised respiratory function as a consequence of impaired ATP synthesis remains the unifying biochemical defect; however the question of how different mutations, particularly those that affect the same enzyme complex, can lead to very different phenotypic presentations remains largely unexplained. It has been proposed that specialised processes within each cell, particularly within neuronal tissue, have a different energy requirement for example, for neurotransmitter release, ion pumping and electrical transmission [34]. In a similar way to tissue-specific dysfunction, cells may be functionally affected in different ways.

Evidence is accumulating for a role of apoptosis-induced cell loss, production of reactive oxygen species and altered calcium metabolism leading to neuronal dysfunction and neuronal degeneration although the relative importance of these three factors remains ambiguous. By identifying the underlying pathogenesis it may be possible to identify therapeutic solutions.

### 4.1 Oxidative stress

Oxidative damage has been implicated as a precursor to apoptosis or programmed cell death. Reactive oxygen species (ROS) are predominantly formed as a by-product of oxidative phosphorylation in the mitochondria [35] with at least 9 submitochondrial ROS-producing sites identified. It is estimated that a significant proportion of oxygen is converted to the superoxide anion during oxidative phosphorylation which is subsequently converted to hydrogen peroxide [36], an important mediator of cellular damage. The enzymes of the respiratory chain are in close proximity to the mtDNA which, in combination with the lack of protective histones, predisposes the genome to mutagenesis. These findings are consistent with studies into pathological mechanisms in specific mtDNA diseases whereby increased ROS production is seen in neuronal NT2 cybrid cells with the LHON mutation. In addition, exogenous administration of antioxidants can protect against ROS-mediated damage in cybrid cells containing the m.8993T>G NARP mutation [37].

There are unfortunately very few models of mtDNA disease in which to explore mechanisms [38]. This is because mtDNA mutations have limited capacity to pass through the germ-line. An alternative approach has been to develop transgenic mice with mutation in the proof reading domain of the mitochondrial polymerase gamma [39,40]. These mice accumulate mtDNA defects and interestingly there was no evidence of increased sensitivity to oxidative stress-induced cell death or of increased levels of ROS ([40,41].

### 4.2 Apoptosis

An increase in the level of ROS or removal of endogenous antioxidants could precipitate apoptosis within a cell. Virtually every cell contains the biochemical participants required to undergo programmed cell death and much research has recently been focused on the extracellular and intracellular pathways that initiate apoptosis. A specific mitochondria-induced pathway has been proposed although mitochondria may be involved in several different pathways. There is some evidence of apoptosis in muscle biopsy samples and cell cultures from patients with specific mtDNA mutations and on autopsy examination neurons are characterised by the shrunken nuclei of apoptosis.

A number of characteristics have focused attention on mitochondria during apoptosis: the early changes of loss of membrane potential, increased porosity of the mitochondrial membrane and the release of proteins from the intermembranous space into the cytosol [42]. One of the key regulators of apoptosis has been identified as the Bcl-2 family of proteins which are located on the mitochondrial membrane. These proteins operate the bulk of control over the release of pro-apoptotic proteins including cytochrome *c* and apoptosis-inducing factor, following which, an apoptosome is formed and the capase cascade is initiated leading terminally to apoptosis [43].

Mitochondria normally co-operate with the endoplasmic reticulum (ER) to ensure homeostasis within the cell. The ER ensures the normal folding of proteins and is a large source of stored calcium ions within the cell which are heavily involved in the transduction of electrical stimulation to molecular response within neurons and have been implicated in the pathways to cell death. The effects of different mitochondrial mutations on mitochondrial and ER initiated apoptotic pathways vary. In some cases the mutations actually appear protective of cell loss [44].

Dysfunction of calcium homeostasis appears to contribute independently to neurodegeneration. Mitochondria have been shown to have an established role in calcium buffering with the capacity to absorb a large amount of calcium rapidly from the cytosol, driven by the large electrochemical gradient across the inner mitochondrial membrane. The calcium is released slowly back into the extracellular space through recycling via the Ca^2+^-ATPase. In mtDNA disease dysfunction may occur due to a decrease in the electrochemical gradient or impairment of the ATP-ases involved in recycling. Increased basal calcium levels and sustained elevation following electrical stimulation have been observed in fibroblasts of patients with the m.3243A>G MELAS mutation.

## 5 Neurological and neurodegenerative features associated with mtDNA mutations (Table 1)

### 5.1 MtDNA point mutations

#### 5.1.1 m.3243A>G — mitochondrial encephalopathy, lactic acidosis and stroke-like episodes (MELAS)

##### 5.1.1.1 Clinical features

The most common presentation of MELAS is a stroke-like episode which often occurs before the age of twenty. Patients typically present on a background of normal psychomotor development, although there may be evidence of short stature. The stroke-like episodes may develop over hours or days and are frequently associated with a severe, migraine-like headache as a prodrome. Seizures are frequently associated and may be present early in the course of the disease, raising the possibility that they could be involved in the pathogenesis. The clinical features of each episode vary according to the location of the lesion and include hemiparesis, focal seizure and cortical visual field loss.

Cortical lesions are observed radiologically and often do not fit with a vascular territory. Diffusion MRI studies have reported a high/normal diffusion coefficient in the presence of MELAS stroke-like episodes compared to the decrease observed in ischaemic lesions. However, a more recent study of serial diffusion MRI scans in one MELAS patient has shown initial restricted diffusion in the context of the acute phase [45]. A restricted diffusion pattern should therefore not exclude MELAS. The radiological lesions may resolve rapidly in keeping with the clinical picture. The pathogenesis of these stroke-like episodes remains controversial.

Intermittent periods of encephalopathy characterised by raised CSF and plasma lactate but not associated with seizures or stroke-like episodes are also seen in patients with m.3243A>G MELAS. A combination of recurrent encephalopathy and stroke-like episodes is thought to cause a slowly progressive neurodegeneration leading in some patients to cognitive decline and dementia [46].

##### 5.1.1.2 MtDNA mutation

The majority of patients (>80%) with MELAS have the m.3243A>G mtDNA point mutation. Patients with this mutation vary from asymptomatic carriers to severely affected individuals with the MELAS syndrome [47]. Patients with the m.3243A>G may also develop diabetes, deafness, ophthalmoplegia (see below), myopathy, gut immobility and cardiac abnormalities stressing the range of different phenotypes seen in patients with mtDNA disease. The m.3243A>G mutation is located in the *MTTL1* gene [48], however, other mutations in this tRNA gene, other tRNA genes and protein encoding genes may also cause the MELAS syndrome [49].

##### 5.1.1.3 Neuropathological studies

**Fig. 1:**
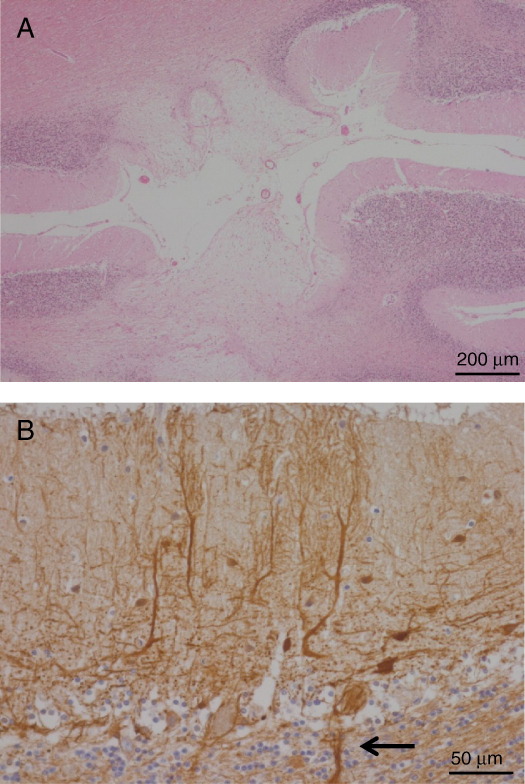
m.3243A>G MELAS mutation: Infarct-like lesion in cerebellar cortex involving the molecular layer, Purkinje cell layer, granular cell layer and white matter (A). There is abnormal dendritic arborisation from remaining Purkinje cells, in vicinity of infarct-like lesion. Swollen axonal terminal synapsing on Purkinje cells shown by arrow (B).

Neuropathological studies have identified infarct-like areas in the white matter of the cortex and subcortex, concentrating principally in the parietal, temporal and occipital lobes [50]. The cerebellum, thalamus and basal ganglia may also be affected [51,52] (Fig. 1). Infarcts are multiple, asymmetrical and, unlike vascular lesions, are not restricted to a particular vascular territory. Microscopically, however, they are similar to true infarcts in both acute and chronic stages. They may be associated with considerable neuron loss, astrogliosis and microvauolation [53]. Ventricular dilatation accompanying cortical atrophy due to extensive neuronal loss is also seen as MELAS progresses.

Calcification of the vasculature of the basal ganglia is also a common neuropathological feature seen in patients with MELAS symdrome and can be identified on neuroimaging [51,54]. Despite this finding the neurones in the basal ganglia are relatively spared and patients rarely present with evidence of basal ganglia dysfunction. The pattern of calcification is similar to that seen in normal ageing and it is possible that the process is simply accelerated by the mitochondrial dysfunction in MELAS.

Extensive damage to the cerebellum is also common in MELAS with loss of Purkinje cells and development of cerebellar ataxia clinically. In severely affected patients cactus formation is visible on Purkinje cells which are thought to be due to the accumulation of abnormal mitochondria within dendrites similar to that seen in ragged red fibres on muscle biopsy.

##### 5.1.1.4 Mechanisms

One theory for the generation of the necrotic lesions in MELAS is the vascular hypothesis which proposes that a deficit in oxidative phosphorylation in cerebral arteries causes an angiopathy which leads to alterations in vascular tone, ischaemia and infarction. This is suggested by the presence of enlarged, abnormal mitochondria in the endothelial and smooth muscle cells of these arteries [55]. Other studies suggest that the proliferation of mitochondria may be a consequence rather than a cause of cerebral ischaemia. Compelling evidence for the vascular hypothesis in MELAS comes from the post-mortem examination of the blood vessels of two patients with MELAS. There was widespread respiratory chain deficiency in the blood vessels and high levels of the m.3243A>G mutation [53]. These results were compared to similar regions from controls which were found to have normal respiratory chain function. The presence of respiratory deficient blood vessels throughout the brain, however, implies that another mechanism must be responsible for dictating the cortical selectivity of brain lesions.

Electron-microscopy has demonstrated abnormal mitochondria within neurones as well as the brain vasculature [56] and data from MR spectroscopic data has shown impaired oxidative metabolism and increased concentrations of lactate in cortical lesions during acute episodes. Observations of impaired glucose uptake in the occipital and temporal regions with positron-emission tomography (PET) in a patient with MELAS may correspond to a higher metabolic rate of neurons in this region [57]. These findings have led to the formulation of the metabolic hypothesis of neurodegeneration in MELAS, which goes some way to explaining the focus of cortical lesion in the parieto-occipital lobes where it is argued there may be a higher metabolic demand on neurones.

#### 5.1.2 8344 A>G — myoclonic epilepsy and ragged red fibres

##### 5.1.2.1 Clinical features

MERRF is a severe neurodegenerative disorder, which often presents in childhood or early adulthood following normal development [47]. The characteristic myoclonus is often the presenting symptom. This progresses into a mixed picture of myopathy, often with pronounced proximal muscle wasting in a limb-girdle distribution; and central neurological features of focal and generalised epilepsy, cerebellar ataxia, optic atrophy, pyramidal signs and hearing loss. A sensory ataxia may also develop from a loss of proprioception due to peripheral sensorimotor neuropathy. The combination of sensory and cerebellar ataxia with myopathy means patients becomes progressively disabled over a period of several decades. Non-neurological manifestations include re-entrant atrio-ventricular tachycardias such as Wolff–Parkinson–White syndrome and multiple lipomas especially in the cervical region. Dysrhythmias can significantly reduce life expectancy with numerous examples of sudden death under the age of 60 in families with a pedigree of MERRF.

##### 5.1.2.2 MtDNA mutation

MERRF is caused most commonly by a point mutation in the *MT-TK* gene at position 8344 in the mitochondrial genome [58]. *MT-TK* encodes the tRNA^Lys^ which is essential for protein synthesis.

##### 5.1.2.3 Neuropathological features

**Fig. 2:**
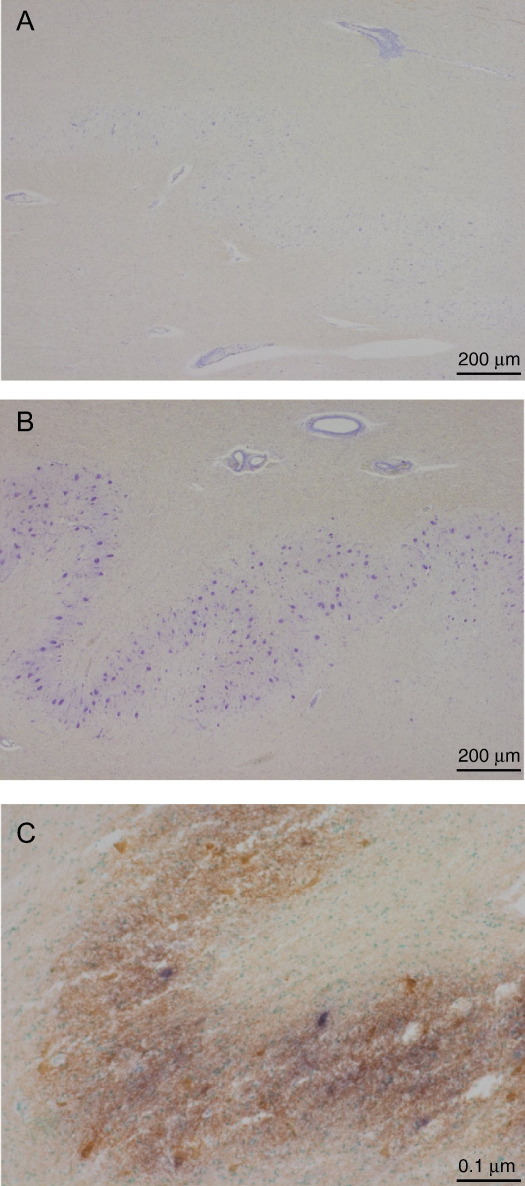
m.8344A>G MERRF mutation: Severe neuronal loss seen in dentate nucleus of patient (A) when compared with age matched control (B). The remaining neurons are often respiratory deficient as shown by blue staining using cytochrome *c* oxidase/succinate dehydrogenase stain (C).

The dentate nucleus is particularly targeted in patients with the m.8433A>G mutation leading to severe neuronal loss accompanied by astrocytosis. Some studies have also demonstrated abnormally large mitochondria with inclusion bodies in this area [59]. An interesting micro-dissection study of a patient with m.8433A>G examined the proportion of mutant mtDNA in different cell types. This identified the vulnerability to this mutation of neurones in the dentate nucleus (Fig. 2) where 45% neuronal loss was noted compared with 7% of cerebellar Purkinje cells. This loss occurred despite the finding that the mutation load in Purkinje cells was 97.6 ± 0.7% compared to 89.0 ± 1.5% in the dentate neurones [60]. Purkinje cell loss is usually mild in MERRF.

The gracile and cuneate nucleus, Clarke's column of the spinal cord, the inferior medullary olives, the pons, and red nuclei of midbrain have all been noted to suffer from some neuronal loss [51]. Although infarct-like areas are found in MERRF as in MELAS, loss of cerebral neurones is rare.

##### 5.1.2.4 Mechanisms

The molecular genetics of the m.8433A>G mutation have been explored *in vitro* with studies of cultured myotubes containing the mutation [61]. In cells containing over 85% mutant impaired protein translation was demonstrated, especially in those proteins with a large number of lysine residues. The authors postulate that the mutation was functionally recessive as a return to 15% wild-type restored translation and respiratory chain activity to near normal activity. Further experiments with rho^0^ cell lines (native mtDNA repopulated with mtDNA containing a specific mutation, in this case the m.8344A>G mutation) have shown that with high levels of mutant load there is a decrease in protein synthesis, oxygen consumption and cytochrome *c* oxidase deficiency.

Muscle biopsy in MERRF shows evidence of mitochondrial accumulations in the sub-sarcolemmal region which gives a characteristic ‘Ragged Red Fibre” when a section of muscle is stained with the Gomori trichrome. The name MERRF was devised before the underlying genetic diagnosis was established and these changes are not specific to patients with MERRF since evidence of mitochondrial proliferation is common in many patients with mitochondrial disease. Mitochondrial defects are now much better detected in muscle using cytochrome *c* oxidase and succinate dehydrogenase histochemistry [62].

#### 5.1.3 m.11778G>A, m.3460G>A and m.14484T>C — Leber's hereditary optic neuropathy

##### 5.1.3.1 Clinical features

Leber's hereditary optic neuropathy (LHON) is an organ-specific disease, targeting the optic nerve. Clinically this presents with a subacute or acute, painless, central visual loss usually between the ages of 20 and 40. Visual loss is typically unilateral with the other eye usually becoming affected within the next two months. Microangiopathy, disk pseudooedema, peripapillary teleangiectasia, tortuous retinal vessels and visual loss ranging from no light perception to 20/60 can all be determined by clinical examination. Whilst the majority of patients with m.11778A>G who have symptoms develop LHON, there have been a few reports of other neurodegenerative phenotypes including an MS like picture and early-onset dystonia [63,64]. The patients with dystonia often do not have visual problems highlighting once again the remarkable clinical variability seen in these diseases. Cardiac conduction defects such as Wolff–Parkinson–White syndrome are also seen in some patients.

##### 5.1.3.2 MtDNA mutation

LHON has a key role in the history of mitochondrial research. First described in 1871 by Thomas Leber as a familial neuro-opthalmologic disease, LHON was the first disease to be recognised as being due to a point mutation in the mitochondrial genome — m.11778G>A [2]. Two other mutations are now also recognised as primary LHON mtDNA mutations m.3460G>A and m.14484T>C. In total these three genes are present in at least 95% of LHON cases [65].

##### 5.1.3.3 Neuropathology

The primary disturbance in patients with LHON is in the retinal ganglion cells and this is reflected in the neuropathology [66]. There is dramatic loss of this layer in the retina as well as the retinal nerve fibre layer, predominantly affecting the central fibres. There is also evidence of on-going neurodegeneration in numerous glial cells and the presence of macrophages filled with lipofuscin.

##### 5.1.3.4 Mechanism

The mutations at m.11778G>A, m.3460G>A and m.14484T>C all involve complex I genes (located in genes MTND4, MTND1 and MTND6 respectively). The progression of disease appears to depend upon the responsible mutation with 71% of patients with the m.14484T>C mutation showing some recovery, compared to 4% in m.11778G>A [67].

The mechanisms proposed for the pathogenesis of these mutations are complex and likely to be multifactorial. The selective vulnerability of the retinal ganglion cells may arise through a combination of bioenergetic failure, oxidative stress, glutamate toxicity, abnormal mitochondrial dynamics and axonal transport and an increased susceptibility to apoptosis [66].

LHON is usually due to a homoplasmic mtDNA mutation; all copies are mutated mtDNA. As such, all maternal offspring will inherit the mutation, however, whilst 50% of males will be affected only 10% of females will develop visual loss. This incomplete penetrance implies a role for nuclear genetic and environmental factors in modulating the expression of the mutation, whilst the male preponderance suggests that there may be an X-linked susceptibility locus [68].

#### 5.1.4 m.8993T>G and m.893T>C — neuropathy, ataxia, and retinitis pigmentosa and Leigh Syndrome

##### 5.1.4.1 Clinical features

Peripheral neuropathy is the principle feature of this disease with other neurological complications including ataxia, retinitis pigmentosa, developmental delay, seizures and dementia. On examination of the patient the neuropathy manifests itself as proximal and distal neurogenic limb weakness, absent ankle jerks and loss of vibration sensation. It has subsequently been recognised that patients with a mutant load greater than 90% have a predominant CNS presentation which usually occurs in infancy [69]. This is known as Maternally Inherited Leigh Syndrome (MILS). As Leigh Syndrome progresses it is associated with stepwise developmental delay followed by developmental regression and death due to respiratory failure. Many other mutations, both mitochondrial DNA and nuclear, can cause Leigh's Syndrome suggesting the developing brain is particularly vulnerable to disturbances of energy metabolism.

##### 5.1.4.2 MtDNA mutation

The m.8993T>G mutation was first described in a single family in which four members had a combination of peripheral neuropathy, ataxia and retinitis pigmentosa [70]. Following this original report, it has also been recognised that a mutation at the same base m.899T>C may also cause the NARP/MILS phenotype. Other mutations of the ATPase 6 gene may also cause the NARP phenotype [71].

##### 5.1.4.3 Neuropathological studies

**Fig. 3:**
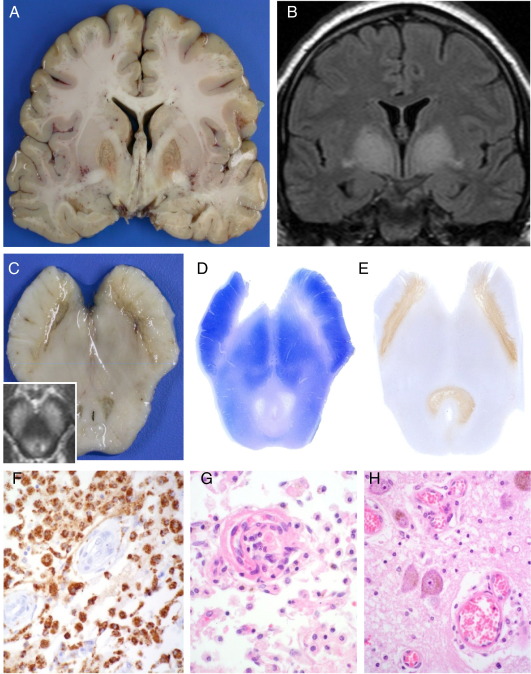
Leigh's syndrome: There is bilateral symmetrical necrosis of the globus pallidus, substantia nigra and periaquaeductal grey matter (A,C) corresponding to areas of increased FLAIR signal three weeks prior to death (B, C inset). Midbrain section stained with Klüver Barrera (D) for myelin and nerve cells, and microglia/macrophage marker CD68 (E) highlights the selectivity of the pathology. (F, G) Increased microvessel density and microvascular proliferation are typical of the fully developed lesion (arrows). (H) Early lesions are characterised by neuropil microvacuolation and vascular ectasia and congestion with preservation of neurons (locus coeruleus) (D, Kluver Barrera; E, F immunohistochemistry for CD68; G, H haematoxylin and eosin; F ×200, G, H ×400).

The neuropathological findings in Leigh's Syndrome are symmetrical, necrotic lesions involving the white and grey matter [72] Fig. 3). The basal ganglia and brainstem are particularly affected and although the clinical picture may be very varied patients often present with symptoms of dysfunction in these areas with respiratory abnormalities, nystagmus, hypotonia and ataxia. Macroscopic examination and neuroimaging demonstrates cystic degeneration particularly in the substantia nigra, periaqueductal grey and putamen. Microscopic changes are more widespread throughout the brain with relative sparing of the cortex and the mammillary bodies. A progression of pathology can be demonstrated with early changes including neuropil oedema and spongy change; capillary proliferation and condensation within or bordering affected white and grey matter, and, tissue rarefaction, which can be severe. Eventually this leads to necrosis. Even in these severely affected areas there is evidence of intact individual neurons which would be unusual in infarcts associated with hypoxia.

##### 5.1.4.4 Mechanisms

The m.8993T>G and m.8993T>C mutations are located within the ATPase 6 gene of the mtDNA. Crucially these mutations do not involve cytochrome *c* oxidase deficiency and as such on muscle biopsy analysis the characteristic ragged red fibres of mitochondrial disease are not present, although there may be evidence of denervation. Studies in blood cells have shown that the m.8993T>G and m.8993T>C mutations both lead to low energy production and ROS overproduction. However, the relative contribution of the two pathogenic components was different; the m.8993T>G change mainly induced an energy deficiency, whereas the 8993T>C favoured an increased ROS production. These results possibly highlight different pathogenic mechanisms generated by the two different mutations at position m.8993.

### 5.2 Single large-scale mtDNA deletions

#### 5.2.1 Chronic progressive external ophthalmoplegia

##### 5.2.1.1 Clinical features

One of the most common presentations of mtDNA disease in adults is chronic progressive external ophthalmoplegia (CPEO). CPEO is characterised by a progressive paralysis of the eye musculature leading to ophthalmoparesis and ptosis. Ptosis is frequently the presenting symptom and may be asymmetrical; however, patients usually progress to bilateral disease. In patients with CPEO there may be other clinical features dependent on the underlying genetic defect, however, myopathy and fatigue are common in all patients.

##### 5.2.1.2 MtDNA mutation

CPEO is typically caused by sporadic large-scale single deletions or multiple mtDNA deletions [73], although in some patients an mtDNA point mutation is detected. Progressive opthalmoplegia also presents as a clinical feature in several other distinct deletion syndromes. A recent meta-analysis attempted to correlate the type of deletion with the phenotype of the patient. It demonstrated that although there is some overlap, the percentage of deletion and the location of the deletion within the genome shows differences between CPEO, Kearns–Sayre Syndrome and Pearson Syndrome (a severe syndrome presenting in infancy with sideroblastic anaemia with pancytopenia and exocrine pancreatic failure) [74].

##### 5.2.1.3 Neuropathological studies

There have been few studies of extraocular muscles in this condition, but skeletal muscle biopsy typically reveals cytochrome *c* oxidase (COX) deficient fibres. Some of these fibres demonstrate characteristic sub-sarcolemmal accumulation of abnormal mitochondria, the classical ragged red fibre. Single muscle fibre analysis has revealed levels of pathogenic mtDNA deletions above a critical threshold level of >80% mutant load in these COX deficient fibres.

##### 5.2.1.4 Mechanisms

The extraocular muscles (EOM) have a wide dynamic range making their structure, biochemistry and immunology distinct from skeletal muscle. This has been suggested as a reason for their selective involvement in certain mitochondrial disorders [75]. A study in 2006 looked at the specific findings in the EOM in patients with CPEO using high-resolution orbital MRI. Unusual signal abnormalities were noted in the EOM with diminished function, particularly in the superior rectus and levator muscles. In these muscles atrophy was also seen, similar to that observed in neurogenic paralysis.

#### 5.2.2 Kearns–Sayre Syndrome

##### 5.2.2.1 Clinical features

Kearns–Sayre Syndrome (KSS) is a rare, progressive mitochondrial disorder which presents with a characteristic triad of: the development of retinitis pigmentosa, progressive external ophthalmoplegia, and occurrence before the age of twenty. Clinical examination in patients with KSS usually detects a ‘salt and pepper’ retinopathy of the posterior fundus without the visual field defects, optic disk pallor and attenuation of retinal vessels usually seen in retinitis pigmentosa. Other neurological complications include cerebellar ataxia, sub-clinical neuropathy, cognitive impairment and deafness. KSS is a multi-system disorder and non-neurological features include cardiomyopathy, complete heart block, short stature, endocrinopathies and dysphagia. Interestingly stroke and epilepsy, which are common in other mitochondrial encephalopathies, are unusual in KSS. On CSF examination patients typically exhibit increased protein and lactate with decreased folate levels.

##### 5.2.2.2 MtDNA mutation

Kearns–Sayre Syndrome (KSS) is typically caused by a large-scale, sporadic single mtDNA deletion, although complex rearrangements can also lead to this disease. No specific deletion is responsible for KSS as all large-scale single deletions will involve multiple protein encoding and tRNA genes. The majority of mtDNA deletions share similar characteristics, with most being located in the major arc between the two proposed origins of replication (O_H_ and O_L_). MtDNA deletions are predominantly (~ 85%) flanked by short direct repeats. About one third of these patients have a 4977 bp deletion, known as the ‘common deletion’ which has a 13 base pair repeat sequence [76].

##### 5.2.2.3 Neuropathological studies

**Fig. 4:**
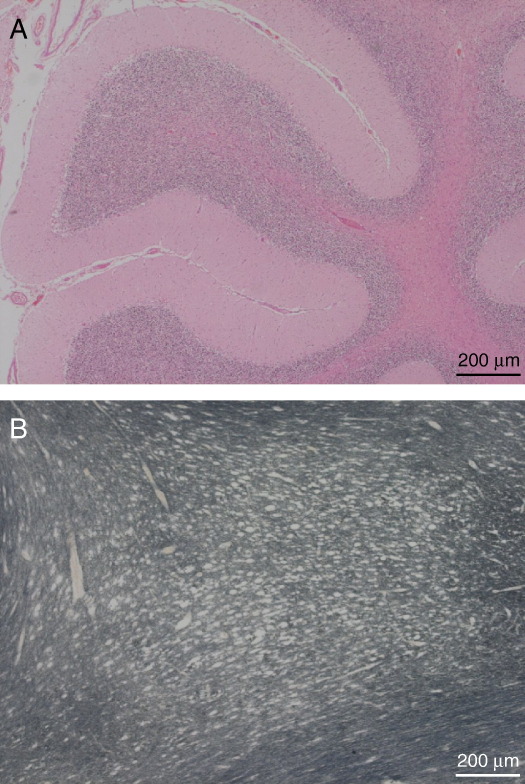
Single large-scale mtDNA deletion: Significant Purkinje cell loss and severe demyelination seen in cerebellum in a patient with KSS (A, B).

The neurodegeneration observed in KSS affects both white and grey matter in the brain (Fig. 4) with widespread spongiform degeneration recognised as a key histological feature of the disease. White matter changes are noted in the cerebrum, cerebellum, thalamus, basal ganglia and spinal cord [51]. The appearance of the white matter depends upon the level of mitochondrial dysfunction and varies from mild atrophy to extensive vacuolation with a sieve-like appearance. Oligodendrocytes appear to be more dependent on optimal mitochondrial activity as they are preferentially affected in KSS. Examination of brain tissue under electron microscope has demonstrated that splits in the intraperiod line are responsible for the spongy appearance of myelin [77].

The grey matter changes and neuronal loss seen in KSS occur predominantly in the cerebellum, the cerebrum and the brainstem with the cortex and the dentate nucleus being relatively spared. The neuropathological findings described are in corroboration with various neuroimaging studies which routinely show diffuse cerebellar, cerebrum and brain stem atrophy. The relative absence of ATP-dependent ion channels and water transporters in astrocytes of the grey matter is thought to be the underlying pathogenic consequence of mitochondrial dysfunction in these areas [78]. To delineate a mechanism for neuronal loss the COX activity of neurones throughout the brain of a patient with KSS were explored. Low levels of COX deficiency were found in the cerebellum, hippocampus, motor cortex and spinal cord with high levels in the reticular formation, nucleus ambiguus and globus pallidus. This inconsistency further highlights the role of a multifactorial neuronal susceptibility to mitochondrial mutations.

Substantial cerebellar degeneration underlies the clinical finding of cerebellar ataxia as the disease progresses. In the cerebellum there is targeted loss of Purkinje cells with development of thicker dendritic trees by the surviving cells apparently in an effort to fill the space. The reason for the restricted depletion of Purkinje cells remains unclear, especially as immunohistochemical analysis appears to suggest a lower threshold for respiratory chain dysfunction within neurones of the dentate nucleus. Other pathological changes include the deposition of calcium in the microvasculature of the globus pallidus and thalamus with haemosiderosis also reported.

##### 5.2.2.4 Mechanisms

A mouse model with a single large-scale deletion, which exhibits many symptoms of KSS may provide us with a better understanding of the pathogenesis of this disease in humans [79]. Currently only skeletal and cardiac muscle have been investigated however it may be possible to examine levels of mutation and COX activity in different populations of neurones at different stages of neurodegeneration. The major mechanistic studies in KSS brains have focused on the changes seen in the choroid plexus. *In situ* hybridisation studies have suggested a possible explanation for the CSF findings in KSS. High levels of deletion have been detected in choroid plexus epithelial cells [78]. It is thought that the mitochondrial dysfunction in these cells leads to an impairment of the blood–brain barrier. This may also play a key role in the pathogenesis of KSS whereby the active transport of folates, which are essential in DNA and RNA synthesis and serotonin metabolism as well as in synthesis of membrane phospholipids, is impaired.

## 6 Potential therapeutic options

Treatment of mtDNA diseases, and in particular the neurological features, remains a major challenge. A review of all therapies [80] revealed little evidence for effective treatment in patients with mtDNA disease. There have been few large clinical trials and unfortunately those that have been performed have shown that medication is either ineffective or toxic [81]. However, supportive therapy is important in managing some of the complications; for example anticonvulsants can control seizures, and diabetes and deafness can be treated with insulin and hearing aids. Cardiac involvement is also common in this group of patients and early management to prevent cardiac failure or to treat cardiac conduction defects is important.

Several groups have been exploring interventions which may increase mitochondrial number or change the mitochondrial genotype. Endurance or resistance exercise is beneficial in patients with improved muscle strength and quality of life [82,83], however, there is no evidence that exercise improves the other neurological features. Recent studies have shown that expression of peroxisome proliferators-activated receptor gamma (PPARgamma), coactivator alpha (PGC1alpha) or administration of bezafibrate (PPARgamma panagonist) induced mitochondrial biogenesis and led to delayed onset of myopathy [84]. Whilst bezafibrate has been used in the treatment of patients with mitochondrial fatty acid defects [85], there have been no published studies in patients with mtDNA disease.

There have been a number of experimental molecular approaches based on either complementing the mtDNA defect or changing the balance of mutated to wild-type mtDNA. To complement mtDNA protein defects it is possible to express the wild-type protein allotopically from nuclear transfected constructs. In cell lines carrying the m.8993T>G NARP mutation, allotropic expression of the wild-type ATPase 6 protein partially rescued the biochemical phenotype [86]. It is also possible to complement mitochondrial tRNA defects. Yeast cytosolic RNA can surprisingly be imported into human mitochondria and it has been shown that the imported tRNA^LysCUU^ can partly rescue the biochemical defect in cell lines carrying the m.8344A>G MERRF mutation [87]. Techniques to shift the mitochondrial genotype have also been investigated including the selective inhibition of mutated mtDNA replication [88] and the import of restriction endonucleases which specifically target the mutated mtDNA [89].

Another approach for the treatment of mitochondrial diseases is expression of single subunit alternative oxidases (AOX) which are found in many eukaryotes but not mammals. These oxidases have the potential to bypass the biochemical defect in oxidative phosphorylation. Recent studies have shown that transgene AOX expression does not produce a detrimental phenotype in Drosophilia and was able to rescue the phenotype two different mitochondrial disease models [90].

Although there is considerable effort to develop treatment for mtDNA diseases, most therapies are a long way from potential clinical use. An alternative approach is to use the unique maternal inheritance pattern of mtDNA diseases to try to prevent the transmission of mtDNA diseases from mother to offspring. Preimplantation genetic testing is of value for some women with heteroplasmic mtDNA defects. Pronuclear transfer in early stage embryos may over a novel approach to preventing transmission of both heteroplasmic and homoplasmic mtDNA disease [91].

## 7 Conclusion

This review has concentrated on describing the clinical and neuropathological features of several different mtDNA diseases. This group of diseases provides some unique challenges to the neuroscientists, not the least because of the very extensive and variable clinical involvement. Understanding the pathogenesis of the neurodegenerative features is challenging because of the lack of good animal models of mtDNA diseases. Whilst there have been animal models of nuclear genes which subsequently affect mtDNA copy number or replication, true inherited mtDNA defect models are difficult to generate. It is difficult to manipulate the mitochondrial genome in cells or animals, and unfortunately pathogenic mtDNA mutations are poorly transmitted through the germ-line.

Very recent epidemiological studies have confirmed that mtDNA disorders are much more common than previously thought, and pathogenic mtDNA mutations are present in about 1 in 200 of the population. This highlights the importance of further studies into the clinical and pathological features of these diseases, particularly as we believe understanding of the mechanisms involved in the neurodegeneration is essential if we are to develop effective treatments for the future. Finally, studying mtDNA diseases may provide important insights for both common neurodegenerative diseases and ageing in which mtDNA defects are also found.

## Figures and Tables

**Table 1:** 

	Genetics	Clinical features	Neuropathology
MELAS (mitochondrial encephalopathy, lactic acidosis, and stroke-like episodes)	mtDNA encoded tRNA and ND gene protein encoding mutations (>80% A3243G in MTTL1 gene)	Stroke-like episodes, lactic acidosis, encephalopathy, myopathy Cardiomyopathy, deafness, diabetes	Multiple, asymmetrical infarcts not restricted to a vascular territory (mainly parietal, temporal, occipital lobes)
			Basal ganglia calcification and cerebellar involvement
MERRF (myoclonic epilepsy and ragged red fibres)	Commonly mtDNA encoded tRNA^Lys^ mutation (m.8344A>G)	Myoclonic epilepsy, cerebellar ataxia, pyramidal signs and hearing loss, optic atrophy	Neuron loss especially dentate nucleus in cerebellum. Less severe involvement of gracile and cuneate nuclei, Clarke's column, inferior medullary olives, red nucleus
		Cardiac conduction abnormalities, lipoma	
LHON (Leber's hereditary optic neuropathy)	m.11778G>A (MTND4)	Optic neuropathy, dystonia	Retinal ganglion cells loss with gliosis
	m.3460G>A (MTND1)	Cardiac conduction defects	
	m.14484T>C (MTND6)		
NARP (neuropathy, ataxia and retinitis pigmentosa)	m.8993T>G and T>C	Peripheral neuropathy, ataxia, retinitis pigmentosa, seizures and dementia	Leigh syndrome: Spongy changes, capillary proliferation and condensation, tissue rarefaction with spared intact neurones. Symmetrical and necrotic lesions involving especially basal ganglia and brainstem
Leigh syndrome		Leigh syndrome (developmental delay and regression, respiratory failure (if mutation load high>90%))	
Single mtDNA deletions	Large-scale single mtDNA deletion	CPEO	KSS
CPEO (chronic progressive external ophthalmoplegia	4977 bp common deletion (1/3 of patients)	CPEO, retinitis pigmentosa, cerebellar ataxia, cognitive impairment, deafness, dysphagia, cardiomyopathy, cardiac conduction defects, short stature, endocrinopathies	White and grey matter spongiform degeneration involving cerebellum, cerebrum and brainstem (sparing of cortex and dentate nucleus)
KSS (Kearns–Sayre Syndrome)			
